# Estimates of global research productivity in primary ovarian insufficiency from 2000 to 2021: Bibliometric analysis

**DOI:** 10.3389/fendo.2022.959905

**Published:** 2022-10-26

**Authors:** Xudong Zhang, Yimeng Lu, Shanshan Wu, Xinyang Zhao, Shuyu Li, Siwen Zhang, Jichun Tan

**Affiliations:** ^1^ Center of Reproductive Medicine, Department of Obstetrics and Gynecology, Shengjing Hospital of China Medical University, Shenyang, China; ^2^ Key Laboratory of Reproductive Dysfunction Disease and Fertility Remodeling of Liaoning Province, Shenyang, China

**Keywords:** premature ovarian insufficiency, POI, POF, Citespace, genetic mutation, stem cell transplantation

## Abstract

**Background:**

Primary ovarian insufficiency (POI) is a heterogeneous disease with diverse clinical phenotypes and etiologies, which is defined as ovarian dysfunction under the age of 40 years. The global prevalence of POI is approximately about 1.1%, and it severely affects female fertility. Nevertheless, bibliometric analysis in this field is extremely limited. We aimed to visualize the research hotspots and trends of POI using bibliometric analysis and tried to predict the future development of this field.

**Methods:**

The original articles regarding POI were culled from the Web of Science Core Collection. Countries, institutions, journals, authors, and keywords in this field were visually analyzed by employing CiteSpace software and Microsoft Excel 2021 software.

**Results:**

A total of 2,999 publications were included for further bibliometric analysis after screening the titles and abstracts stringently. The number of literature regarding POI significantly increased yearly. These publications come from 78 countries. The USA was dominant in the field of POI in terms of the number of publications (865), average citations per item (57.36), and *h*-index (112). The *Institut National De La Sante Et De La Recherche Medicale Inserm* is the most high-yield institution in this field with 351 publications. *Fertility and Sterility* ranked first with the highest number of publications (152), followed by *Human Reproduction* (138). According to the keyword cluster analysis from 2000 to 2021, the eight keyword clusters encountered frequently were apoptosis, osteoporosis, fertility preservation, mutation, fragile x syndrome, adrenal insufficiency, DNA repair, ovarian reserve. Keyword citation burst analysis revealed that whole-exome sequencing, ovarian tissue cryopreservation, and DNA repair had a citation burst until 2021.

**Conclusions:**

Great progress has been made in POI research over the past 20 years, which is widely researched but unevenly developed in the world. In terms of influence, the United States may be in the lead. The research hotspots in POI are mainly pathogenesis and treatment, including genetic mutation, hormone therapy, fertility preservation, and stem cell transplantation.

## 1 Introduction

Bibliometric analysis is a world-accepted statistical evaluation of published articles and has grown in popularity, which was first proposed by American bibliographers in 1969. Through qualitative and quantitative analyses of publications, it could use literature metrology characteristics to provide investigators with crucial messages, discover frontiers, and evaluate the distribution of countries/regions, authors, and journals in a certain specific field (1). Primary ovarian insufficiency (POI) was first documented in 1942 and has been described with different names since then, such as premature ovarian insufficiency, premature/primary ovarian failure (POF), and premature menopause (2). Although a significant body of literature has been published related to POI, these publications have not been summarized and analyzed.

POI is a heterogeneous condition with diverse clinical phenotypes and etiology, which is defined as the development of amenorrhoea due to loss of ovarian activity under the age of 40 years (3). The global prevalence of POI is approximately 1.1% (3), which appears to vary among different ethnic populations. The diagnosis is based on elevated FSH levels in the menopausal range (usually above 40 IU/l) detected on at least two occasions more than a month apart (4). The pathological features of POI are follicle dysfunction (follicles remain but not functioning normally) and follicle depletion (no primordial follicles remain) (5). It is characterized by elevated levels of gonadotropins and low levels of estradiol (6), accompanied by a series of symptoms including reduction in ovarian function and primary or secondary amenorrhea/oligomenorrhea. Hypoestrogenism could result in menstrual irregularities and a myriad of menopausal symptoms, such as hot flashes, night sweats, insomnia, and even anxiety disorder. Moreover, the long-term consequence of POI is an increased lifetime risk of cardiovascular disease (7), osteoporosis (8), earlier mortality, and neurocognitive disorders (9).

The etiology is unknown in 70%–90% of diagnosed patients (5), so POI is usually identified as a spontaneous or idiopathic disease. One of the early recognized causes is the presence of autoimmune syndromes, which is approximately 20% of POI cases (10). Furthermore, anticancer treatments (surgery, chemotherapy, or radiation) may severely damage ovarian function to generate POI in women with various malignancies (11). Genetic mutations and chromosomal abnormalities are now deemed to play a more significant role (12–14). In addition, it is reported that environmental impacts and lifestyle factors also play a part (15).

Hormone replacement therapy (HRT) is one of the most commonly used treatments (16), which is required to relieve hypoestrogenism symptoms and prevent the long-term health sequel of estrogen deficiency. However, the majority of patients with POI are seeking fertility; conventional hormone therapy alone cannot restore ovarian functions fundamentally. Thus, it is urgent to seek novel treatments to improve fertility, such as stem-cell transplantation, *in vitro* activation (IVA), and platelet-rich plasma (PRP) infusion (17), that could solve the disorders of ovaries from the root and offer tremendous potential in treating POI in the future.

In this study, we aimed to provide a general description of quantitative and visual information on the research of POI, identifying its emerging trends and potential hotspots through an integrative analysis of relevant information from manuscripts published worldwide from 2000 to 2021. We presented a brief discussion of POI research and predicted possible trends in this field in the next few years, providing the groundwork for future research directions and developments.

## 2 Methods

### 2.1 Research strategy and data extraction

The data were extracted from the Web of Science Core Collection (WoSCC, Clarivate Analytics), reserving any bias arising from the constant updating of the database. The search formula was set to (TS = “premature ovarian insufficiency” OR “primary ovarian insufficiency” OR “premature menopause” OR “premature ovarian failure” OR “POI” OR “POF”) through the “Topic” retrieval approach, and the time span was set from 1 January 2000 to 31 December 2021 based on when the research on POI began to rise. The publication type was limited to original articles, whereas languages other than English were excluded. Then, we removed papers that were irrelevant to POI by screening the titles and abstracts carefully.

The final result was agreed on after a series of search processes by Yimeng Lu and Xudong Zhang. Both researchers independently reviewed each article for inclusion according to the preset criterion. Given that data were directly downloaded from the database, ethics was not required.

### 2.2 Bibliometric analysis

A range of key information was extracted from the articles for further analysis, such as title, author, research institution, country/region, keywords, year of publication, source, number of citations, 2020 impact factor (IF), and the Hirsch index (*h*-index). Articles originating from England, Scotland, Northern Ireland, and Wales were reclassified as from the United Kingdom (UK). IF was derived from the 2021 Journal Citation Reports (JCR) (Clarivate Analytics, Philadelphia, United States). The *h*-index was defined as a certain country or institution that has published h papers each cited at least h times (18) and was calculated as a qualitative measure to assess the scientific research performed in the field of POI for the top-ranked authors, countries, and institutions.

Firstly, Microsoft Excel 2021 was used for quantitative and qualitative analyses to clarify the general information of publications to generate relevant charts and graphs. The assessment includes (I) the research trends in the POI field based on the total citations each year (from 2000 to 2021); (II) the top 25 authors based on the number of publications, total citations, and *h*-index; and (III–V) the top 10 countries, institutions, and journals based on the total publications in retrieved publications.

Secondly, the annual number of publications per country was analyzed using the Literature Metrology Online Analysis Platform. The world map was generated online (https://www.mapchart.net/index.html).

Thirdly, CiteSpace (5.8.R3) (19) was used to analyze the information of authors, institutions, journals, countries, and other factors; plot the relevant visual networks; and predict the research prospect and hotspots. CiteSpace also facilitated the extraction of a certain number of papers within a specified number of years into a single network through the “time-slicing” function. We chose different node types, and the numbers of publications and citations were represented by their sizes. The parameters of CiteSpace were set as follows: method (LLR), time slicing (2000–2021), years per slice (1), term source (all selection), node type (choose one at a time); selection criteria (g-index: k = 25), and minimum duration (MD = 2).

## 3 Results

**Figure 1:**
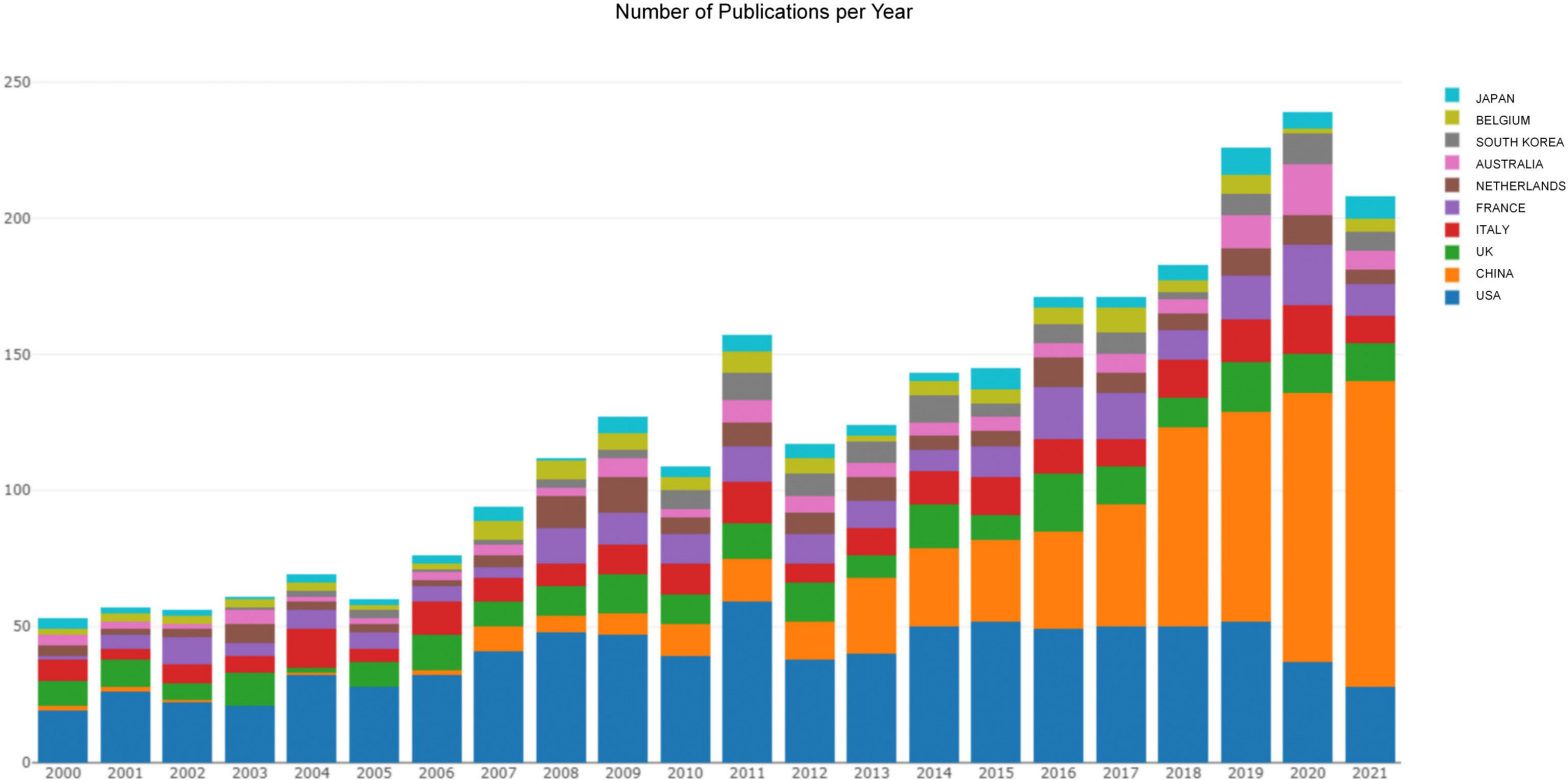
The publishing performance of countries. The yearly production trend per country in the field of POI published from 2000 to 2021.

Searching the WoSCC database, we retrieved a total of 11,284 records from 2000 to 2021. After screening titles and abstracts stringently to exclude unrelated papers and duplicate studies, 2,999 articles were included for further bibliometric analysis finally. Overall, the yearly production trend increased steadily and showed two clear peaks, one in 2011 (n = 166) and the other in 2020 (262). However, there was a slight decrease in 2021 (n = 221) (**Figure 1**).

**Figure 2:**
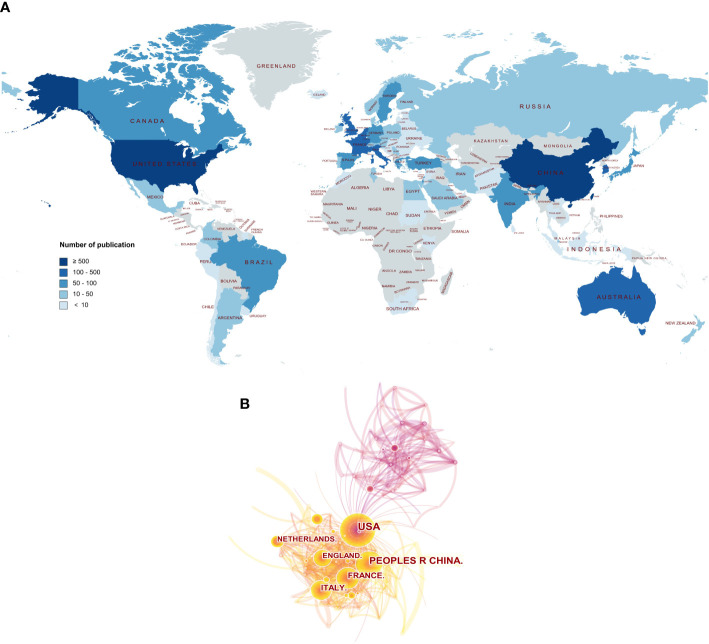
The publishing performance of countries. **(A)** The worldwide view of the research output of countries. The color intensity represents the number of publications. The world map was generated online (https://www.mapchart.net/index.html). **(B)** The collaboration between the countries. Each node represents a country, and the number of publications was reflected by the node size.

A total of 78 countries participated in POI research across the world. The view of the research output of countries across the globe is displayed in **Figure 2A**. The contributions in this field are mainly concerned in Asia, Europe, North America, and Oceania, whereas Africa contributes the lowest proportion of publications. As shown in **Table 1**, the top 10 countries were ranked by *h*-index. The highest *h*-index was achieved by the USA (112), followed by the UK (64), Italy (56), France (56), and China (50). These countries contributed to 92% of all research publications in the field of POI between 2000 and 2021. The USA was the predominant country (28.8%), followed by China (20.1%), the UK (8.4%), Italy (7.8%), and France (7.7%). Belgium had the highest average citations per item (ACI) value (90.95), followed by the USA (57.36), the UK (55.91), and the Netherlands (54.14). **Figure 1** shows the annual number of publications per country and the USA (blue) is on a steady trend, whereas China (orange) is rapidly growing. The collaboration between the countries is shown in **Figure 2B**. The USA took the core position in international collaboration in this field, which was the main partner of the other productive countries.

**Table 1:** Top 10 productive countries regarding the research on POI in terms of the *h*-index.

Country	*h*-index	Quantity	Percentage	ACI
USA	112	865	28.8%	57.36
UK	64	252	8.4%	55.91
Italy	56	234	7.8%	50.31
France	56	230	7.7%	44.57
China	50	604	20.1%	18
Netherlands	49	146	4.9%	54.14
Belgium	47	102	3.4%	90.95
Australia	39	122	4.1%	48.96
Japan	31	97	3.2%	46.82
South Korea	24	107	3.4%	18.84

ACI, average citations per item.

Among the top 10 institutions rank by *h*-index in **Table 2**, four institutions were from France, three institutions were from the USA, two institutions were from China, and one institution was from Belgium. The *Udice French Research Universities* led first with the highest *h*-index (50), followed by the *National Institutes of Health NIH USA* (47), the *Assistance Publique Hopitaux Paris Aphp* (44), the *Universite De Paris* (44), and the *Institut National De La Sante Et De La Recherche Medicale Inserm* (43). Moreover, the *Udice French Research Universities* also achieved the highest institutional productivity (165). The *Harvard University* had the highest ACI (96.46).

**Table 2:** Top 10 institutions in the studies of POI rank in order by *h*-index.

Institution	*h*-index	Quantity	Percentage	ACI
*Udice French Research Universities*	50	165	5.5%	43.6
*National Institutes of Health NIH USA*	47	120	4.0%	63.23
*Assistance Publique Hopitaux Paris Aphp*	44	122	4.1%	45.52
*Universite De Paris*	44	106	3.5%	47.8
*Institut National De La Sante Et De La Recherche Medicale Inserm*	43	125	4.2%	41.03
*University of California System*	42	109	3.6%	60.39
*Harvard University*	39	78	2.6%	96.46
*University of London*	36	75	2.5%	60.68
*Shanghai Jiao Tong University*	25	80	2.7%	25.14
*Shandong University*	23	87	2.9%	30.33

ACI, average citations per item.

**Figure 3:**
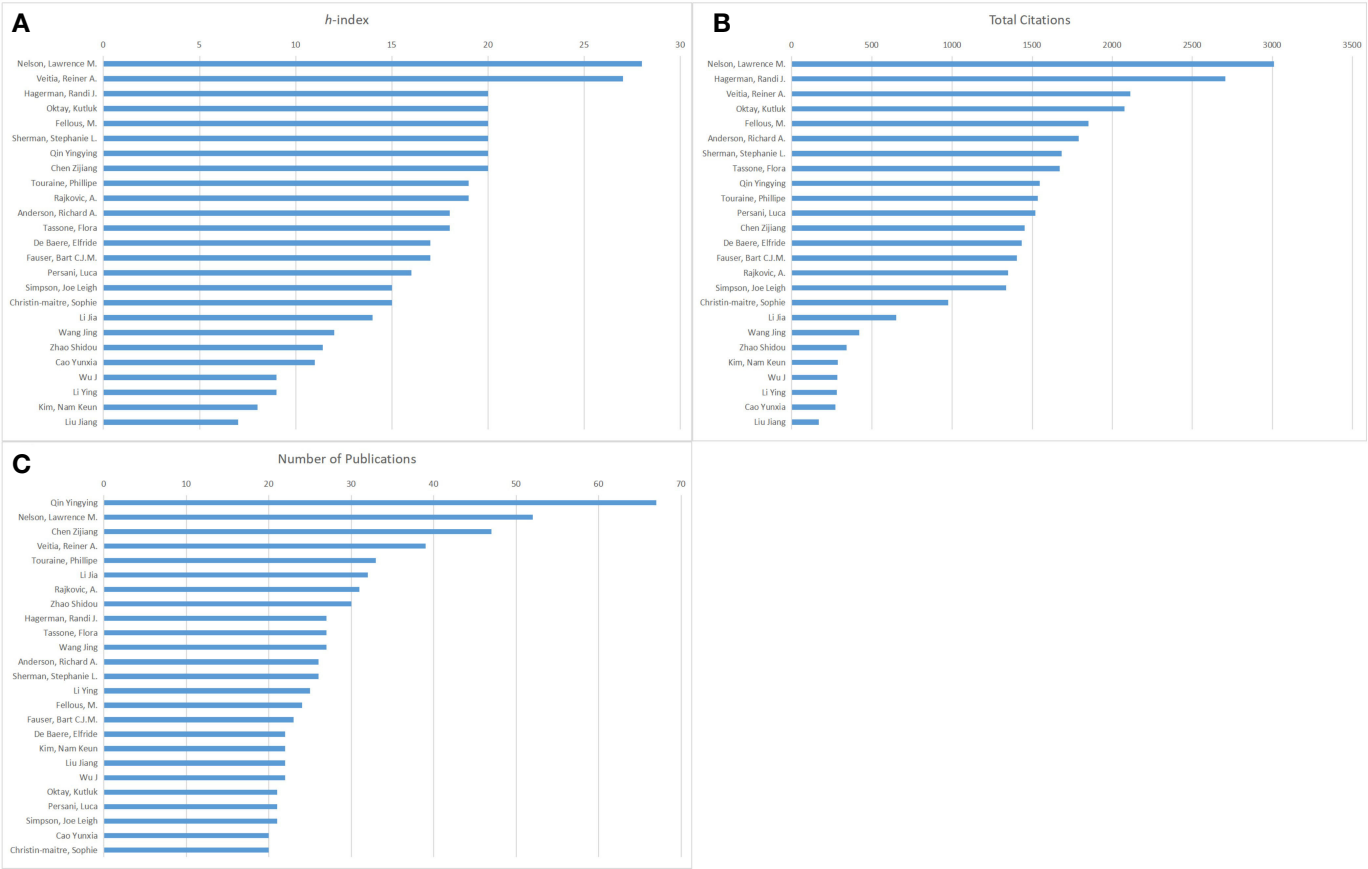
The publishing performance of authors. **(A)** The top 25 authors based on the *h*-index from 2000 to 2021. **(B)** The top 25 authors based on total citations from 2000 to 2021. **(C)**The top 25 authors based on the number of publications from 2000 to 2021.

**Figure 3** presents the top 25 authors based on the *h*-index, total citations, and number of publications in this field. These authors have published at least 20 articles during the period of study. Nelson Lawrence M. ranked first based on the *h*-index (28) and total citations (3009) (**Figures 3A, B**). Veitia Reiner A. placed second (27) in **Figure 3A**. Hagerman Randi J., Oktay Kutluk, Fellous M., Sherman Stephanie L., Qin Yingying, and Chen Zijiang have the same *h*-index (20). In **Figure 3B**, Hagerman Randi J. placed second (2706), followed by Veitia Reiner A. (2112), Oktay Kutluk (2077), and Fellous M. (1850). As shown in **Figure 3C**, Qin Yingying had the highest number of publications with 67 articles, followed by Nelson Lawrence M. (52), Chen Zijiang (47), and Veitia Reiner A. (39).

**Table 3** summarizes the top 10 journals rank by impact factors (IF). *Fertility and Sterility* ranked first with IF of 7.490, followed by *Human Reproduction* (6.353), *Journal of Clinical Endocrinology & Metabolism* (6.133), *Maturitas* (5.110), and *Reproductive Biomedicine Online* (4.567). According to the JCR 2021, these 10 most active journals were spread across Q1, Q2, and Q3. The top 10 journals contributed 25.04% of the overall publication output (751/2,999). The total citations of these 10 journals ranged from 1,284 to 117,818.

**Table 3:** Top 10 journals in the studies of POI rank in order by IF.

Journal	IF	TP	TC
*Fertility and Sterility*	7.490	152	115715
*Human Reproduction*	6.353	138	117818
*Journal of Clinical Endocrinology* & *Metabolism*	6.133	77	117612
*Maturitas*	5.110	40	112983
*Reproductive Biomedicine Online*	4.567	66	1284
*Journal of Assisted Reproduction and Genetics*	3.357	52	94109
*Menopause-The Journal of the North American Menopause Society*	3.310	67	111333
*Climacteric*	3.024	49	64465
*Gynecological Endocrinology*	2.277	69	115689
*Seminars in Reproductive Medicine*	1.912	41	117547

IF, impact factors; TP, total publications; TC, total citations.

**Table 4** lists the top 10 cited articles related to POI research, including information on author, title, source, year of publication, total citations, and PMID. The total citations of the 10 most cited articles varied from 545 to 1,178. The most highly cited article was authored by Coates et al. and published in *Annals of Oncology* in 2015, entitled “Tailoring therapies-improving the management of early breast cancer: St Gallen International Expert Consensus on the Primary Therapy of Early Breast Cancer 2015”.

**Table 4:** Top 10 citation analysis of documents on POI.

Authors	Title	Source title	Cited by	Year of publication	PMID
Coates, A. S.	Tailoring therapies-improving the management of early breast cancer: St Gallen International Expert Consensus on the Primary Therapy of Early Breast Cancer 2015 (20)	*Annals of Oncology*	1178	2015	25939896
Donnez, J	Livebirth after orthotopic transplantation of cryopreserved ovarian tissue (21)	*Lancet*	1157	2004	15488215
Velde, ERT	The variability of female reproductive ageing (22)	*Human Reproduction Update*	770	2002	12099629
Crisponi, L	The putative forkhead transcription factor FOXL2 is mutated in blepharophimosis/ptosis/epicanthus inversus syndrome (23)	*Nature Genetics*	756	2001	11175783
Castrillon, DH	Suppression of ovarian follicle activation in mice by the transcription factor Foxo3a (24)	*Science*	667	2003	12855809
Nelson, Lawrence M.	Primary Ovarian Insufficiency (5)	*New England Journal of Medicine*	628	2009	19196677
Uhlenhaut, N. Henriette;	Somatic Sex Reprogramming of Adult Ovaries to Testes by FOXL2 Ablation (25)	*Cell*	621	2009	20005806
Bondy, Carolyn A.	Clinical practice guideline - Care of girls and women with Turner syndrome: A guideline of the Turner Syndrome Study Group (26)	*Journal of Clinical Endocrinology* & *Metabolism*	621	2007	17047017
Meirow, D	The effects of radiotherapy and chemotherapy on female reproduction (27)	*Human Reproduction Update*	575	2001	11727861
Reddy, Pradeep	Oocyte-specific deletion of Pten causes premature activation of the primordial follicle pool (28)	*Science*	545	2008	18239123

**Figure 4:**
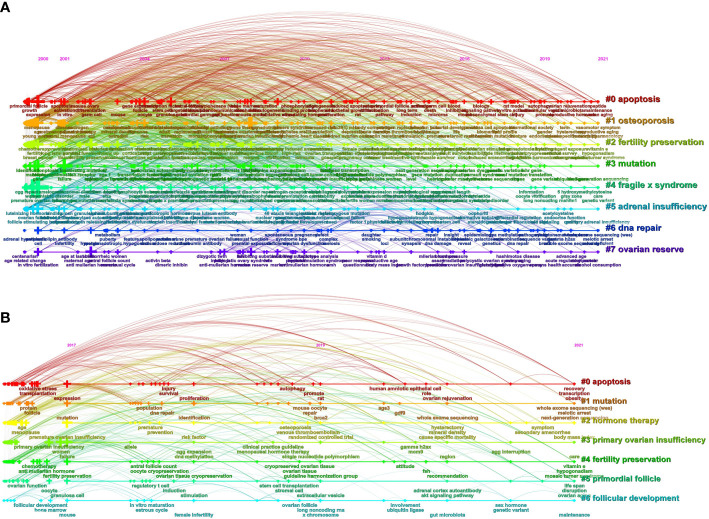
The publishing performance of keywords. **(A)** Timeline view of keyword clusters on POI research from 2000 to 2021. **(B)** Timeline view of keyword clusters on POI research in the past 5 years. The horizontal line shows the clusters. The crosses on the horizontal line represent the keywords contained in the corresponding cluster, and their size represents the frequency of keywords. The curved line represents the relationships between the clusters. The cluster labels are on the right.

**Figure 5:**
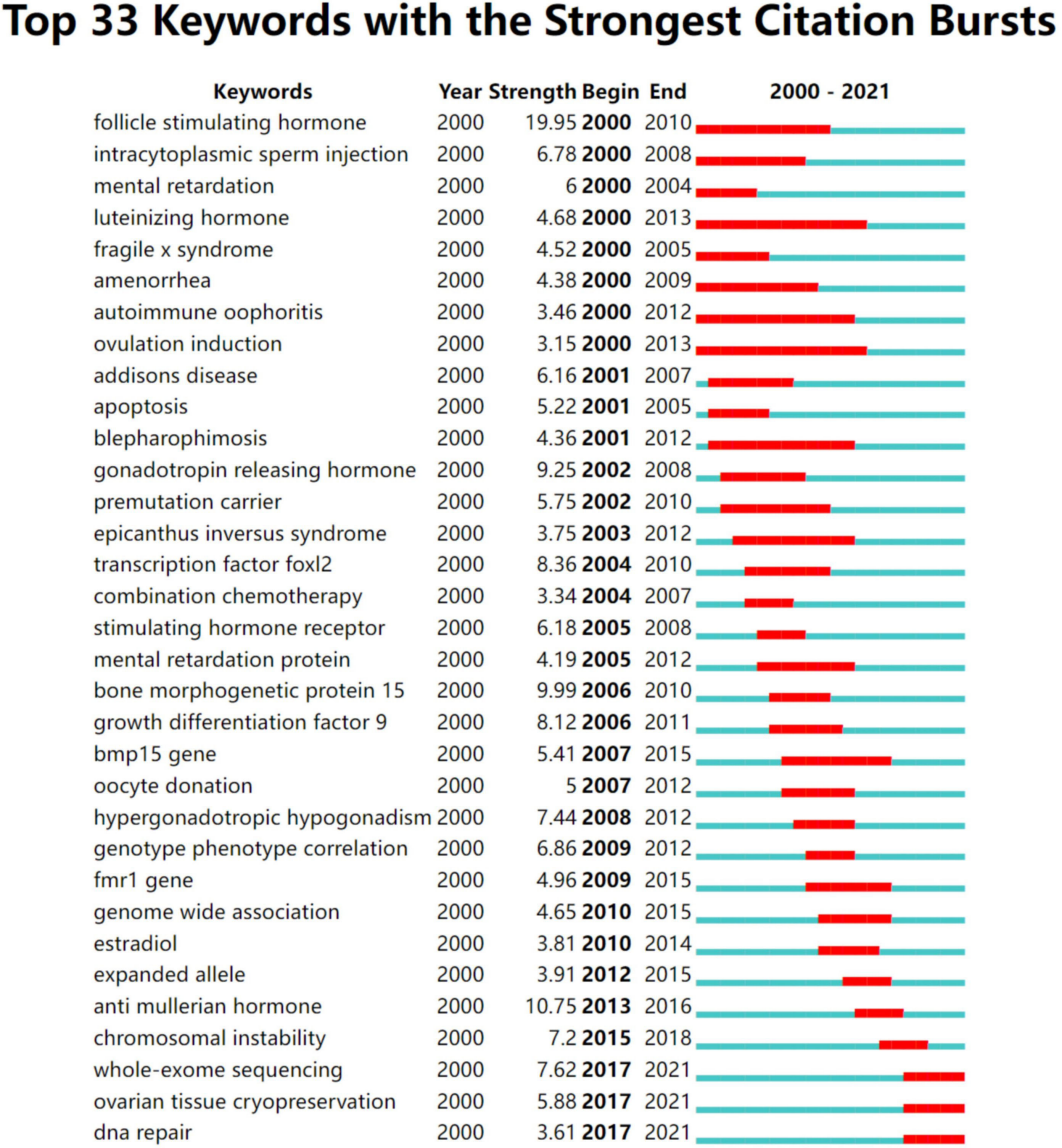
The publishing performance of keywords. Keywords with the strongest citation bursts in publications on POI research from 2000 to 2021. The timeline is depicted as a year-sliced blue line, with the period when a subject was observed to have a burst marked as a red selection.

The keywords extracted from titles and abstracts highlight the main focus of the research presented in a scientific document. **Figure 4A** shows a timeline view of keyword clusters formed by the most frequently encountered topics in the title or/and abstracts of all retrieved publications. Eight clusters were identified, namely, #0 apoptosis; #1 osteoporosis; #2 fertility preservation; #3 mutation; #4 fragile x syndrome; #5 adrenal insufficiency; #6 DNA repair; #7 ovarian reserve. Four clusters were related to pathogenesis (#0, #3, #4, #5, and #6), two clusters were related to phenotype (#1 and #7), and the other cluster was related to treatment (#2). In order to further understand the research hotspots in the past 5 years, we conducted a timeline view of keywords clusters from 2017 to 2021 (**Figure 4B**). Seven clusters were identified, namely, #0 apoptosis; #1 mutation; #2 hormone therapy; #3 primary ovarian insufficiency; #4 fertility preservation; #5 primordial follicle; #6 follicular development. Similar to **Figure 4A**, these seven clusters are mainly related to the pathogenesis (#0 and #1) and treatment (#2 and #4). **Figure 5** shows the top 33 representative keywords with the strongest citation bursts. The strength of “whole-exome sequencing,” “ovarian tissue cryopreservation,” and “DNA repair” was 7.62, 5.88, and 3.61, respectively, which are currently within the burst period (to 2021).

## 4 Discussion

This is a comprehensive bibliometric analysis that we have known in the field of POI, which involved 2,999 original articles retrieved from WoSCC. We have assessed the POI research from multiple perspectives, such as yearly publications and total citations, publication outputs of countries, institutions, and authors with their total citations and *h*-index, journals with their IF, and cluster analysis of keywords, to identify global trends and research hotspots related to POI.

The yearly production is on a steady upward trend in this field. The USA ranked first in terms of the number of publications, citations, *h*-index, and the number of internationally collaborative publications. Moreover, we can find that most of the high-yield countries are located in Asia, Europe, North America, and Oceania, whereas Africa contributes the lowest proportion of publications (**Figure 2A**). Academic achievements may be inseparable from a country’s economic development, scientific progress, and infrastructure construction. As shown in **Figure 1**, the annual number of publications in China has exponentially increased recently. The total number of publications in China is second-highest after the USA (**Table 1**). Undoubtedly, China has made great efforts in this field, especially in the past decade. According to the European Society for Human Reproduction and Embryology (ESHRE) guideline, the global prevalence of POI is approximately 1.1% (3), which appears to vary among different ethnic populations. In 2004, a large-scale population-based cohort study in Shanghai Women’s Health reported that the prevalence of POI is 2.8% in Chinese women (29). A recent study showed that about 4 million reproductive-age women in China suffer from POI, which may be one of the reasons for the increasing research productivity in China in this field (30).

Hot research topics are presented by the common co-occurrence of terms in the title or abstract, obtained by the timeline view of keywords cluster analysis, which provides a substantial and valuable insight into which topics are motivating the research field over time (31). An analysis of the keywords could be used as a quantitative index to identify the most important topics and trends in different research fields (32). Over these years (2000–2021), significant progress has been made in the field of POI research. In general, the research hotspots in POI involve pathogenesis and treatment (**Figures 4, 5**). The POI genetic spectrum has been expanded remarkably with the development of sequencing techniques, such as next-generation sequencing (NGS) and whole exome sequencing (WES). Therefore, the study of genetic mutation in POI patients is a research hotspot of POI. Currently, the clinical treatment of POI is mainly concentrated on hormone therapy used as a general routine therapy, ovarian tissue cryopreservation used as fertility preservation for cancer patients, and stem cell transplantation used as a novel strategy.

### 4.1 The pathogenesis of POI

POI is still identified as an idiopathic disease in most cases. The etiologies of POI mainly include genetic defects, iatrogenic causes (surgery, chemotherapy, or radiation-induced), and autoimmunity.

Genetic factors are regarded as a strong component of the pathogenesis of POI since POI is a highly heterogeneous disease. Genetic factors mainly involve chromosomal abnormalities and genovariation (33, 34). Chromosomal abnormalities have been recognized as one of the common causes of POI, which mainly affect the X chromosome but also the autosome. Both numerical abnormalities and terminal deletion of the X chromosome can cause POI (35). Some studies suggest that approximately 12% of cases of genetic POI are induced by errors with the X chromosome gene complement (36, 37). Turner syndrome is the only viable monosomy condition in humans with only one X chromosome (38). One of the most frequently noted phenotypes of Turner syndrome is POI, which had been recognized as the most established genetic cause of POI. Accumulative studies have concluded that such infertility associated with Turner syndrome results from premature apoptosis of germ cells (39). Furthermore, the huge leap in genetic knowledge associated with the pathogenesis of POI was obtained by next-generation sequencing (NGS) and whole-exome sequencing (WES). NGS is a method of simultaneously sequencing millions of fragments of DNA, whereas WES is a novel method derived from NGS, which detects all exons of all human genes and is suitable for the diagnosis of complex genetic diseases that are difficult to determine (40). Some possible candidate genes associated with POI have been found by sequencing, including *FMR1* (41, 42), *FOXL2* (23, 43), *FOXO3A* (24), *BMP-15* (44, 45), *FSHR* (46, 47), *POF1B* (48), *NR5A1* (49), *FIGLA* (50), *GDF-9* (51), *BRCA1* (52), *PGRMC1* (53), *NABOX* (54, 55), and *MCM* (56, 57). These genes may promote the development of POI by inducing apoptosis of ovary cells, primordial follicles, and granulosa cells (58), suppressing ovarian follicles activation (24), and motivating follicle atresia (59).

Advancements in anticancer treatment have led to improvements in long-term survival but led to infertility and POI at the same time since the ovarian follicles are extremely susceptible to the effects of chemotherapy and radiotherapy (27). Compared with the general population, women patients are 38% less likely to have a pregnancy after cancer treatment (60). The gonadotoxic degree of chemotherapy is largely drug and dose-dependent (61–63). Alkylating agents are a typical type of gonadotoxic agents, such as cyclophosphamide and anthracycline (63). Total body, cranial, whole abdominal, or pelvic irradiation potentially may sustain permanent damage to the ovary and loss of primordial follicles, resulting in POI. The degree of impairment is strongly related to total radiation dose, volume treated, and age (64).

Autoimmunity is responsible for approximately 4%–30% of POI cases (10, 65). Evidence involves the presence of anti-adrenal antibodies, the histological appearance of lymphocytic oophoritis, and coexistence with other autoimmune disorders (66, 67). Autoimmune POI may be associated with adrenal autoimmunity, non-adrenal autoimmunity, or isolation. Adrenal autoimmune POI is the most frequent type, especially Addison’s disease-related POI (AD-POI) (68, 69). A clinical study which included 258 women with (AD) showed that 20.2% of these patients had POI, and 72% of patients with AD-POI detected steroid-producing cell antibodies (StCA), which may be a marker of POI in patients with AD (70).

### 4.2 The treatments of POI

#### 4.2.1 Hormonal replacement therapy

The main agents of HRT are estrogens and progestogens. Patients with a history of hysterectomy are recommended to take estrogen-alone therapy, whereas those with an intact uterus should use combining estrogen-progestogen therapy (71). Progestogens could reduce the risk of endometrial hyperplasia and carcinoma that may occur with estrogens (72).

The goal of HRT in patients with POI is to restore normal serum estrogen concentrations. HRT is strongly recommended in women with POI, except where contraindicated. HRT can not only treat the symptoms of low estrogen but also relieve the symptoms of vasomotor and genitourinary (16) and prevent bone loss and cardiovascular disease (73, 74). Patients with POI require long-term HRT; thus, natural or near-natural estrogens and progestogens are recommended to reduce the adverse effects on the breast, metabolism, and cardiovascular. However, studies have shown the increased risk of breast cancer with combining estrogen-progestogen therapy, which might be associated with the dose and duration of use, whereas the risk is significantly lower with estrogen-alone therapy (75). HRT should be initiated as early as possible based on no contraindications and careful evaluation.

#### 4.2.2 Infertility treatment

Infertility is an important and urgent problem for patients with POI. A study showed that intermittent ovulation may occur in 24% of idiopathic women with POI (76). Although intermittent ovarian activity in women with POI exists, pregnancy rates are low. The most effective treatment for POI patients is oocyte donation (OD); however, many of these women with infertility prefer to use their gametes rather than OD upon the initial diagnostic interview, requesting alternative solutions despite the low odds of success (77).

The main cause of infertility is a absence of the pool of primordial follicles in patients with POI; the antral follicle count is usually low, and the response to ovarian stimulation is poor. A meta-analysis comparing the gonadotropin-releasing hormone (GnRH) agonist protocol with the GnRH antagonist protocol in poor responders showed no significant difference in live birth rates (78). However, significantly fewer oocytes were retrieved in the GnRH antagonist protocol group (79, 80). Currently, the most widely used gonadotropins (Gn) are highly human menopausal gonadotrophin (hMG) and recombinant FSH (rFSH) (81). The use of rFSH and hMG for ovarian stimulation in GnRH agonist is recommended (82). One suggested strategy proposed recently was a double-stimulation protocol (follicular and luteal) in the same ovarian cycle, especially for those patients with a significantly reduced antral follicle count that Gn stimulation cannot function well (83).

Given that some patients whose antral follicle counts are too low to achieve the success of *in vitro* fertilization (IVF), *in vitro* activation (IVA) and fresh ovarian tissue autotransplantation can be used to obtain competent and healthy fertilizable oocytes from residual follicles. Traditional IVA combined two methods. One is using PTEN enzyme inhibitors and PI3K activators to enhance the AKT pathway to awake the “dormant” follicles in ovaries (84). Another is the fragmentation of the ovaria cortex to interfere with the Hippo pathway and restore ovarian follicle growth (85). However, it has been suggested that activation by pharmacological methods may negatively affect the quality of oocytes (86). Recently, a modification of IVA (drug-free IVA) has been proposed to disrupt the Hippo-signaling pathway alone without using chemical drugs. A prospective observational cohort study showed that follicle development was detected in half of the patients (7/14) and oocyte retrieval was achieved successfully in 5/14 patients by using drug-free IVA (87). In clinical application, drug-free IVA could be a new useful therapeutic option for patients with POI, through the combination of IVF technology, to help patients get their offspring.

Iatrogenic factors (chemotherapy, radiotherapy, or surgery) have become a vital cause in the pathogenesis of POI, and protection against iatrogenic POI continues to be an important clinical problem. The practice of fertility preservation (FP) in women with cancer is spreading. A study investigated the long-term reproductive outcomes in patients with breast cancer who underwent FP compared with no history of FP, and the result showed that live birth rates among women receiving FP were significantly higher (22.8% vs. 8.7%) (88). In 2006, the American Society of Clinical Oncology (ASCO) proposed that shield or ovarian transposition during radiotherapy, fertility-sparing surgery, GnRH analogue administration during chemotherapy, and embryo cryopreservation after ovarian stimulation with gonadotrophins and IVF should be considered in young patients (89). Cryopreservation of oocytes and embryos after controlled ovarian stimulation is the standard strategy for FP in clinics (90). Intact embryos after thawing have similar implantation potential as fresh embryos, which can lead to a 59% pregnancy rate and a 26% live birth rate (91). Moreover, the pregnancy rate and live birth rate after fertilization of frozen-thawed oocytes are currently reaching those obtained after embryo cryopreservation (92). Due to the limitation that both oocyte cryopreservation and embryo cryopreservation require ovarian stimulation, ovarian tissue cryopreservation (OTC) can be considered. OTC does not require ovarian stimulation, which avoids a significant delayed initiation of cancer treatments and could be performed in prepubertal children (93). A meta-analysis reported live birth and ongoing pregnancy rates of 37.7% after receiving ovarian tissue cryopreservation and transplantation (94).

Recently, autologous platelet-rich plasma (PRP) has been reported for women with POI receiving IVF (95, 96). PRP is a plasma fraction of autologous blood with a high concentration of platelets, which have been suggested to be able to enhance angiogenesis, tissue regeneration, and the cell proliferation process (97, 98). PRP has been proven to support the viability and growth of human early pre-antral follicles (99) and increase the number of follicles and oocytes by intraovarian injection directly under transvaginal sonographic guidance (100). The implantation rates and the live birth rates are better in patients who have received intraovarian PRP infusion (101).

#### 4.2.3 Stem cell therapy

Nowadays, stem cell-based regenerative medicine holds great promise for the restoration of non-functional tissues or organs. Numerous pieces of research pointed out the efficacy of stem cell transplantation in POI treatment, and a series of clinical trials have been conducted to prove its safety and effectiveness accordingly. Stem cells are used to regenerate the ovarian niche to promote the development of the remaining follicles in the ovary (102). Various types of stem cells have been already used to treat POI with the development of regeneration medicine. Among them, mesenchymal stem cells (MSCs) are the most widely used, which have various sources, including bone marrow, adipose tissue, amniotic fluid, amniotic membrane, placenta, menstrual blood, endometrium, and umbilical cord (103–107). A clinical trial has shown that umbilical cord-derived MSC transplantation can improve the development of follicles and increase the number of oocytes retrieved in women with POI, and all patients showed good clinical outcomes without side effects or complications (108). Recent studies have further proposed that the underlying mechanism of MSCs may be the numerous extracellular vesicles (EVs) secreted by them, which contain a variety of proteins and genetic materials. A study by Zhang et al. demonstrated that EVs from menstrual blood-derived MSCs are effective in restoring ovarian functions and fertility in POI through *in vitro* and *in vivo* research (109).

In summary, POI is still highly heterogeneous in etiology and phenotype. It is difficult for clinical doctors to take effective and optimal treatments in time. It will seriously affect patients’ physical and mental health and further influence the stability of their families. Therefore, the priority is to comprehend as much as possible about the etiology to guide treatment. The expansion of sequencing techniques is expected to enable the discovery of new causative genes of POI. However, most existing studies on novel genes carried out were limited and only to some specific groups. Thus, it is necessary to perform genetic research on populations with large sample sizes and across different ethnic populations. According to the ESHRE Guideline Group on POI (3), patients with POI should undergo a routine screening, including chromosomal analysis and autoimmune antibody analysis (ACA/21OH, thyroid antibodies, and TPO-Ab). The optimal approaches to management are considering not only to promote fertility but also to control certain factors related to the development of POI. However, the most common therapies focus on treating the symptoms without addressing the root cause of the problem currently. Hence, in the future, a more novel and secure therapy may be expected to cure POI.

### 4.3 Strengths and limitations

This is an extensive bibliometric analysis study including publications over 20 years (2000–2021) and thus can investigate the global POI research trends more credibly. In this study, we extracted the publications from the emergence of research on POI to the present and evaluated findings from an overall point of view; thus, we could realize the primary messages and core progress in the field of POI. However, this study still exerts some limitations. First, the language of papers was restricted to English, which result in that some studies written in other languages may have been omitted. Second, only the WoS was searched to collect data due to the limitation of the CiteSpace software, and some other databases such as Scopus and Google Scholar, which might ensure a better representation of the available academic outputs in this field, were not analyzed. Third, due to time constraints of publications, the citations of newly published literature are lower, leading to being overlooked.

## 5 Conclusion

The present work of this study was to conduct a global bibliometric analysis of POI research from 2000 to 2021. POI holds great research significance in women’s reproductive health. Tremendous strides have been made in POI research currently. The POI genetic spectrum has been expanded remarkably with the development of sequencing techniques, such as NGS and WES. These possible candidate genes influence a variety of biological activities, including hormonal signaling, metabolism, DNA repair, and immune function. Although curing POI remains a challenge, a substantial amount of research has been produced; it is promising to foresee that some novel strategies can be widely used to combat POI fundamentally in the future.

## Data availability statement

The original contributions presented in the study are included in the article/supplementary material. Further inquiries can be directed to the corresponding author.

## Author contributions

YL and XZhang was responsible for experiment conception and design, collection and assembly of data, data analysis and interpretation,and manuscript writing. SW contributed to acquisition data and revised the manuscript. XZhao, SL, and SZ contributed to the revision of the manuscript. JT designed the work, provided technical guidance, and finally approved the manuscript. All authors read and approved the final manuscript.

## Funding

This work was supported by the Key Research and Development Program of Shenyang (20-205-4-011), the National Natural Science Foundation of China (82071601), the Key Research and Development Program of Liaoning Province (2018225093), the Major Special Construction Plan for Discipline Construction Project of China Medical University (3110118033), and the Shengjing Freelance Researcher Plan of Shengjing Hospital of China Medical University.

## Conflict of interest

The authors declare that the research was conducted in the absence of any commercial or financial relationships that could be construed as a potential conflict of interest.

## Publisher’s note

All claims expressed in this article are solely those of the authors and do not necessarily represent those of their affiliated organizations, or those of the publisher, the editors and the reviewers. Any product that may be evaluated in this article, or claim that may be made by its manufacturer, is not guaranteed or endorsed by the publisher.
